# Physical Activity Patterns and Neighborhood Characteristics of First-Generation Latina Immigrants Living in Arizona: Cross-sectional Study

**DOI:** 10.2196/25663

**Published:** 2021-05-17

**Authors:** Rodney P Joseph, Sonia Vega-López, SeungYong Han

**Affiliations:** 1 Center for Health Promotion and Disease Prevention Edson College of Nursing and Health Innovation Arizona State University Phoenix, AZ United States; 2 College of Health Solutions Arizona State University Phoenix, AZ United States; 3 Southwest Interdisciplinary Research Center Arizona State University Phoenix, AZ United States; 4 Edson College of Nursing and Health Innovation Arizona State University Phoenix, AZ United States

**Keywords:** emigrants and immigrants, physical activity, exercise, residence characteristics, female, metabolic disease, Latina, immigrants, emigrants, health outcomes

## Abstract

**Background:**

Metabolic diseases, including obesity and type 2 diabetes, are a major health concern for Latina immigrants. Performing regular aerobic physical activity (PA) is a lifestyle behavior associated with the prevention and control of these conditions. However, PA levels of most Latina immigrants are below national guidelines. Neighborhood environmental factors may influence the PA levels of adults, but limited research has explored associations between the neighborhood environment and PA levels among Latina immigrants.

**Objective:**

The objective of this study was to explore the PA patterns of first-generation US Latina immigrants and how neighborhood environmental factors are related to those PA patterns.

**Methods:**

Using a cross-sectional study design, 50 first-generation Latina immigrants completed the International Physical Activity Questionnaire (IPAQ) and the Neighborhood Scales Questionnaire, which assessed 6 perceived neighborhood factors: (1) walking environment, (2) aesthetic quality, (3) safety, (4) violence, (5) social cohesion, and (6) activities with neighbors. Median self-reported metabolic equivalent (MET)-minutes/week of PA were used to summarize domain-specific (ie, work, domestic/household, leisure, and transportation) and intensity-specific (ie, walking, moderate, vigorous, moderate to vigorous) PA patterns. Logistic regression examined associations between neighborhood factors and engaging in leisure-time PA (ie, dichotomous outcome of some versus no leisure-time PA), transportation PA (ie, dichotomous outcome of some versus no transportation PA), and meeting national PA guidelines (ie, dichotomous outcome of meeting versus not meeting guidelines).

**Results:**

Preliminary analyses showed that 10 participants reported excessively high PA levels and 1 participant had incomplete PA data; these women were excluded from analyses based on IPAQ scoring guidelines. The remaining 39 participants (mean age 40.5 years; mean length of US residency 4.6 years) reported a median of 4512 MET-minutes/week of total PA. The majority of PA was acquired through domestic activities (median 2160 MET-minutes/week), followed by leisure-time PA (median 396 MET-minutes/week), transportation PA (median 198 MET-minutes/week), and work PA (0 MET-minutes/week). Intensity-specific PA patterns showed a median of 594 MET-minutes/week of walking activity and 3500 MET-minutes/week of moderate-to-vigorous PA. Logistic regression models indicated that the neighborhood factors of walking environment, aesthetic quality, and safety were positively associated with engaging in leisure-time PA (odds ratios of 5.95, 95% CI 1.49-23.74; 2.45, 95% CI 1.01-5.93; and 3.30, 95% CI 1.26-8.67, respectively) and meeting national PA guidelines (odds ratios of 4.15, 95% CI 1.13-15.18; 6.43, 95% CI 1.45-28.39; and 2.53, 95% CI 1.00-6.36, respectively). The neighborhood factors of violence, social cohesion, and activities with neighbors were not significantly associated with PA outcomes.

**Conclusions:**

Although most participants met national PA guidelines (ie, ≥500 MET-minutes/week of moderate-to-vigorous PA), the majority of their PA was achieved through domestic activities, with limited leisure, transportation, and work PA. Given that leisure-time PA in particular plays a significant role in improving health outcomes, findings suggest that many Latina immigrants could benefit from a leisure-time PA intervention. Such interventions should consider neighborhood environmental influences, as these factors may serve as determinants of PA.

## Introduction

Metabolic disease conditions are a major health concern for Latina immigrants. Findings from the Hispanic Community Health Study/Study of Latinos (N=16,415) indicate that 45% of first-generation Latina immigrants (ie, Latinas born outside of the United States) are obese [1] and 17% have type 2 diabetes [2]. In comparison, national surveys estimate the prevalence of these conditions as 38% and 7%, respectively, among non-Latina White women and 40% and 9% among the US population as a whole [3,4]. Important to the discussion on the prevalence of metabolic diseases among Latina immigrants is the pattern in which they develop. Upon initial entry into the United States, Latina immigrants have an equal-to-lower risk prevalence of obesity and diabetes compared with US-born Latinas and non-Latina Whites [5-7]. However, as duration of US residency increases, so does risk for developing obesity and diabetes conditions [2,5-10].

The high metabolic disease burden among Latina immigrants represents a major public health concern. Latinx immigrants are the largest immigrant group in the United States, accounting for approximately 40% of the total US immigrant population, and are projected to remain the largest immigrant population through at least the year 2055 [11]. Thus, a better understanding of lifestyle behaviors that may contribute to the high metabolic disease prevalence among first-generation Latina immigrants with longer duration of US residency is essential to developing interventions to address this public health concern.

Regular aerobic physical activity (PA) is an independent lifestyle behavior associated with the prevention and control of both obesity [12-14] and diabetes [15]. However, the PA levels of most Latina immigrants are below the national guidelines of 150 minutes/week of moderate-intensity PA [16-18]; data on this population are limited due to the limited number of studies that differentiate by generational status. Existing data also show a decline in PA as duration of US residency increases [19]. This decline in PA parallels the increase in metabolic disease risk observed among this population of US Latinas. Interventions designed to promote sustained high levels of PA may be key in addressing the metabolic disease prevalence among Latina immigrants.

The development of effective PA interventions requires researchers to have extensive understanding of the PA patterns of Latina immigrants as well as the social and environmental factors that may influence PA behaviors. The purpose of this study was to explore the PA patterns of first-generation Latina immigrants and the neighborhood environments in which they reside in the metropolitan area of Phoenix, Arizona. This research was conducted as part of a formative process to inform the development of culturally tailored PA interventions for first-generation Latina immigrants. First, we explored domain-specific PA engagement (ie, occupational, household, transportation, and leisure-time PA). Given the limited research on this topic among recently immigrated first-generation Latinas [16,18,20] and emerging evidence suggesting that domain-specific PA differentially influences health outcomes (ie, high levels of leisure-time PA have the most profound impact on improving health outcomes [21-23], while high levels of occupational PA may have a limited-to-negative effect on improving health outcomes [21,24-26]), such research is necessary to gain an understanding of the PA patterns of recently immigrated US Latinas. Second, we examined the social and physical characteristics of the neighborhoods in which recently immigrated Latinas reside. We were interested in neighborhood characteristics because most people spend the majority of their nonworking time in or around their residential neighborhood. Thus, the neighborhood characteristics of Latina immigrants are likely to influence the types of PA promoted through a PA intervention [16,18,20].

## Methods

### Study Design, Setting, and Participant Recruitment

A cross-sectional study design with self-report survey data was used. Data were collected as part of a broader study examining how immigration and integration experiences of recently immigrated first-generation Latinas influence perceptions of and opportunities for PA. Participants were a convenience sample of Latinas recruited from the metropolitan area of Phoenix. Recruitment strategies included flyer advertisements and in-person recruitment at predominately Hispanic-serving community outreach centers, health clinics, religious institutions, and local businesses. The Phoenix metropolitan area includes the cities of Phoenix, Mesa, and Chandler and is predominately comprised of urban and suburban neighborhoods. The region has an estimated 4.8 million residents, with 30% (ie, 1.5 million) identifying as Hispanic or Latinx [27]. Among residents identifying as Hispanic or Latinx, 400,000 are foreign-born and approximately 60,000 immigrated to the United States after 2010 [27]. Women were eligible for participation in this study if they (1) self-identified as Latina, (2) immigrated into the United States within the past 10 years, (3) were aged ≥18 years, and (4) self-reported an ability to read in English or Spanish. Only women immigrating into the United States within the previous 10 years were included because the purpose of the larger study from which data were collected was to explore how contemporary social and contextual factors, including the impact of more restrictive immigration policies enacted during the US presidency of Donald Trump, were related to PA engagement among recently immigrated Latinas.

Women interested in study participation completed an eligibility screening interview either in-person or via telephone (according to the recruitment method). Eligible women were then given the option to complete the study questionnaire packet in English, Spanish, or a combined English/Spanish language using a completion method of their choice. Methods of survey completion included online via Qualtrics or by an oral interview. All participants were provided with an informed consent document prior to completing the survey. Participants completing the survey online provided informed consent by selecting the “continue” button on the webpage displaying the informed consent document that was accompanied by the following statement: “By clicking the Continue button, I acknowledge that I am at least 18 years old and that I consent to conducting the survey online.” Verbal informed consent was obtained from participants completing the survey via the oral interview or pencil-and-paper format. After completing the survey, participants were provided a US $25 gift card for study participation. All study procedures were approved by the Institutional Review Board of Arizona State University.

### Measures

#### Demographics

Participant demographic characteristics were collected using a form developed for this study. Age, country of origin, and years of residence in the United States were obtained using the following open-ended questions: “How old are you?”, “Where were you born?”, and “How long have you been living in the United States?” Characteristics of primary language at home, monthly household income, education level, marital status, and employment status were asked using closed-ended questions modeled after items used in the 2017 Behavioral Risk Factor Surveillance System survey [28]. All demographic questions are available in Multimedia Appendix 1.

#### Duration and Frequency of PA

PA was assessed using the long version of the International Physical Activity Questionnaire (IPAQ) [29]. This 27-item questionnaire, previously validated in both English and Spanish [29,30], asks respondents the duration (minutes/week) and frequency (days/week) of PA that they engage in according to 5 domains: (1) work, (2) transportation, (3) domestic/household, (4) leisure time, and (5) sitting. Responses were used to estimate the minutes/week that participants engaged in each activity. Self-reported minutes/week of activity were then weighted based on estimated energy expenditure (ie, metabolic equivalents [METs]) to provide an estimate of total weekly PA volume (ie, MET-minutes/week), as well as an estimate of energy expenditure for time spent in domain- and intensity-specific (ie, walking, moderate, and vigorous intensity) activities. When respondents indicated that they did not perform activity in a given domain, the value for that domain was set to zero (eg, if a participant indicated that she did not perform paid or unpaid work outside of the home, the value for work-related PA was set to 0). All data were scored and reported according to guidelines published in 2005 [31] and were calculated as a continuous measure of MET-minutes/week.

#### Neighborhood Environment

The neighborhood environmental factors of walking environment, aesthetic quality, safety, violence, social cohesion, and activities with neighbors were assessed using the Neighborhood Scales Questionnaire [32]. Scales measuring neighborhood walking environment (7 items), aesthetic quality (5 items), safety (3 items), and social cohesion (4 items) have respondents rate their agreement with various statements using a 5-point Likert scale (ie, 1=strongly agree, 2=agree, 3=neutral, 4=disagree, and 5=strongly disagree). Example statements include “I often see other people walking in my neighborhood” (from the walking environment scale) and “I feel safe walking in my neighborhood, day or night” (from the safety scale). Scales assessing neighborhood violence (4 items) and activities with neighbors (4 items) have participants rate the frequency of specific events on a 4-point Likert scale (1=often, 2=sometimes, 3=rarely, and 4=never). Example items from these scales include “During the past 6 months, how often was there a fight in your neighborhood in which a weapon was used?” (from the violence scale) and “How often do you and other people in your neighborhood visit in each other’s homes or speak with each other on the street?” (from the “activities with neighbors” scale). All scales were scored individually by calculating the mean score of individual scale items. For ease of data interpretation, scores were reverse ordered, when appropriate, to indicate that higher scores are associated with higher walkability, aesthetic quality, safety, social cohesion, violence, and activities with neighbors. The Neighborhood Scales Questionnaire was developed and validated in both English and Spanish [32] and has established test-retest reliability (ie, intraclass correlation coefficients ranging from .60 to .88) [32]. Internal consistency estimates (ie, Cronbach alpha coefficients) for the scales ranged from .78 to .92 in this study, which are comparable to previous research [32,33].

### Statistical Analysis

Descriptive statistics (ie, means, 95% confidence intervals, medians, interquartile ranges, and frequencies) were used to summarize participant demographic, PA, and neighborhood variables. A series of regression models were used to examine associations between neighborhood environmental factors and the PA outcomes of leisure-time PA, transportation PA, and overall moderate-to-vigorous PA. Associations between neighborhood factors and work and household PA were not examined because we lacked a theoretical rationale for why neighborhood factors would influence these PA variables. Ordinal least squares (OLS) regression models, controlling for age and level of education, were used to examine the independent effect of each neighborhood factor on continuous PA outcomes in MET-minutes/week. Logistic regression, controlling for age, was used to examine associations between neighborhood factors and dichotomous PA outcomes of engaging in leisure-time PA (ie, engaging in 0 versus >0 MET-minutes/week of leisure-time PA), transportation PA (ie, engaging in 0 versus >0 MET-minutes/week of transportation PA), and meeting national PA guidelines (ie, engaging in <500 versus ≥500 MET-minutes/week of moderate-to-vigorous PA). Level of education as a control variable was not included in logistic models because of perfect prediction. As a result of the collinearity of neighborhood variables, associations between each neighborhood variable (ie, walking environment, aesthetic quality, safety, violence, social cohesion, and activities with neighbors) and PA outcomes were examined separately. Stata/SE version 16.0 (StataCorp) was used for data analysis.

## Results

### Participants

A total of 50 first-generation Latinas participated in the study. However, preliminary data cleaning revealed that 10 participants reported unreasonably high PA data (ie, the sum of all walking, moderate, and vigorous PA time was greater than 960 minutes or 16 hours/day) and 1 participant had incomplete IPAQ data. These women were excluded from data analysis according to IPAQ scoring guidelines [31], resulting in a final sample size of 39 participants. Sensitivity analyses (ie, chi-square test for categorical variables and *t* test for age and duration of US residency) were conducted to explore demographic differences between participants excluded from outcome analyses (n=11) and those included. Results showed that the women excluded from the study had a lower education level than those included (*P*=.003). No other demographic differences were observed between women included in the study and those excluded.

Among the 39 women included in outcome analyses, the mean age was 40.5 (SD 4.3) years and the mean duration of US residence was 4.6 (SD 1.0) years. The majority of the participants were from Mexico (27/39, 69%), with the remaining participants from various Central and South America countries. Approximately half (22/39, 56%) of the women were married and the majority spoke Spanish exclusively at home (32/39, 82%). Based on data provided by the IPAQ, only 7 (18%) of the 39 participants reported performing paid or unpaid work outside of the home. Complete demographic characteristics are presented in Table 1.

**Table 1:** Demographic characteristics of study participants included in the outcome analysis (N=39).

Characteristic			Value
Age (years), mean (95% CI)			40.5 (36.2-44.8)
Age (years), median (minimum; maximum)			39.0 (2.4; 78.0)
Duration in the United States (months), mean (95% CI)			55.6 (43.5-67.7)
Duration in the United States (months), median (minimum; maximum)			52.0 (5.0; 128.0)
**Country of origin, n (%)**			
	Mexico	27 (69)	
	Venezuela	5 (13)	
	Colombia	4 (10)	
	Cuba	1 (3)	
	Dominican Republic	1 (3)	
	Nicaragua	1 (3)	
**Primary language spoken at home, n (%)**			
	English	1 (3)	
	English and Spanish	6 (15)	
	Spanish	32 (82)	
**Monthly household income, n (%)**			
	≤$1000	4 (10)	
	$1001-$2000	13 (33)	
	$2001-$3000	7 (18)	
	$3001-$4000	6 (15)	
	>$4000	2 (5)	
	Don’t know	4 (10)	
	Refused to respond	3 (8)	
**Education, n (%)**			
	Elementary	2 (5)	
	Middle school	2 (5)	
	High school	6 (15)	
	Some college or university	11 (28)	
	University graduate or postgraduate	18 (46)	
**Marital status, n (%)**			
	Single or no partner	6 (15)	
	Married	22 (56)	
	Cohabiting	3 (8)	
	Separated	1 (3)	
	Divorced	2 (5)	
	Widowed	5 (13)	
**Employment, n (%)**			
	Unemployed/looking for work	3 (8)	
	Unemployed/not looking for work	1 (3)	
	Homemaker	12 (31)	
	Student	1 (3)	
	Retired	2 (5)	
	Other	2 (5)	
	Missing	18 (46)	

### PA Outcomes

Table 2 provides a detailed overview of intensity- and domain-specific PA patterns of our sample. Participants reported a median of 4512 MET-minutes/week of total PA. Intensity-specific PA outcomes showed that the majority of this PA was achieved through moderate-intensity activity (median 2820 MET-minutes/week). Median self-reported MET-minutes/week of walking and vigorous-intensity PA were 594 and 0, respectively. Overall, 85% (33/39) of participants reported PA levels that met the requirements of the PA guidelines (ie, ≥500 MET-minutes/week of moderate-to-vigorous PA).

**Table 2:** Summary statistics of intensity- and domain-specific metabolic equivalent (MET)-minutes/week of physical activity (PA).

			MET-minutes/week of PA	
Outcome		Participants, n (%)	Mean (95% CI)	Median (IQR)
Total PA^a^		39 (100)	6128 (4277-7979)	4512 (7271)
**PA by intensity**				
	Walking	39 (100)	963 (532-1394)	594 (1386)
	Moderate	39 (100)	3842 (2696-4988)	2820 (4410)
	Vigorous	39 (100)	1323 (545-2101)	0 (1440)
	Moderate to vigorous^b^	39 (100)	5165 (3456 -6874)	3500 (6060)
**Achieved PA guidelines^c^**				
	Met PA guidelines	33 (85)	236 (18-454)	270 (396)
	Did not meet PA guidelines	6 (15)	7199 (5225-9174)	5040 (7778)
**Work PA**				
	Participants reporting working outside of the home	7 (18)	6009 (1171-10,846)	4590 (10,536)
	Work PA—full sample^d^	39 (100)	1079 (65-2092)	0 (0)
**Transportation PA**				
	Participants reporting transportation PA	23 (59)	584 (365-803)	360 (594)
	Transportation PA—full sample^d^	39 (100)	345 (188-501)	198 (453)
**Domestic/household PA**				
	Participations reporting domestic/household PA	34 (87)	3514 (2497-4531)	2850 (4024)
	Domestic/household PA—full sample^d^	39 (100)	3063 (2102-4025)	2160 (4560)
**Leisure-time PA**				
	Participants reporting engaging in leisure-time PA	26 (67)	24623 (1146-3779)	1042 (2997)
	Leisure-time PA—full sample^d^	39 (100)	1642 (704-2580)	396 (1980)
**Sitting time**				
	Weekday total	38 (97)	333 (267-399)	300 (240)
	Weekend total	39 (100)	327 (250-405)	240 (240)

^a^The sum of walking and moderate and vigorous PA, which is equal to the sum of work, transportation, domestic/household, and leisure-time PA domains.

^b^The sum of moderate and vigorous PA.

^c^Achieving PA guidelines was defined as engaging in ≥500 MET-minutes/week of moderate-to-vigorous PA; not meeting PA guidelines was defined as engaging in <500 MET-minutes/week of moderate-to-vigorous PA.

^d^Participants reporting that they did not engage in a domain-specific activity had their value for that given domain set to zero; full-sample values for each domain include these participants with their zero values and thus provide an overall summary statistic for the domain that includes all study participants.

Examination of domain-specific PA patterns showed that domestic/household PA was the most frequent type of PA reported by participants (34/39, 87%) and, on average, how participants acquired the majority of their activity (ie, median 2160 MET-minutes/week). Leisure-time PA was the second most frequently reported domain of activity (26/39, 67%), followed by transportation (23/39, 59%) and work (7/39, 18%) PA. Additionally, although only 7 participants reported working outside of the home according to the IPAQ, the amount of PA acquired by these participants through work activity was higher than any other domain (4590 MET-minutes/week; see Table 2).

### Associations Between Neighborhood Environmental Factors and PA

Table 3 shows the descriptive values for scales assessing neighborhood environmental factors. OLS regression results, presented in Table 4, revealed no significant associations between neighborhood environmental factors and continuous PA measures. Table 5 presents logistic regression models examining associations between neighborhood environmental factors and dichotomous PA outcomes of engaging in transportation PA versus not engaging in transportation PA, engaging in leisure-time PA versus not engaging in leisure-time PA, and meeting PA guidelines versus not meeting PA guidelines. Results showed that the neighborhood factors of walking environment, aesthetic quality, and safety were significantly associated with engaging in leisure-time PA (OR 5.95, 95% CI 1.49 to 23.74; OR 2.45, 95% CI 1.01 to 5.93; and OR 3.30, 95% CI 1.26 to 8.67, respectively) and meeting national PA guidelines (OR 4.15, 95% CI 1.13 to 15.18; OR 6.43, 95% CI 1.15 to 28.39; and OR 2.53, 95% CI 1.00 to 6.36, respectively). No other significant associations were found.

**Table 3:** Summary statistics of perceived neighborhood factors.

	Neighborhood Scales Questionnaire [32] scale scores		
Perceived neighborhood factor	Observations, n	Mean (95% CI)	Median (minimum; maximum)
Walking environment^a^	38	3.9 (3.6-4.1)	3.9 (1.9; 5.0)
Aesthetic quality^a^	39	3.7 (3.4-4.0)	3.8 (1.0; 5.0)
Safety^a^	38	3.4 (3.1-3.7)	3.7 (1.0; 5.0)
Social cohesion^a^	35	3.1 (2.8-3.4)	3.3 (1.0; 5.0)
Violence^b^	33	1.4 (1.2-1.7)	1.0 (1.0; 5.0)
Activities with neighbors^b^	38	1.7 (1.5-1.9)	1.6 (1.0; 3.4)

^a^Score range of 1 to 5.

^b^Score range of 1 to 4.

**Table 4:** Ordinal least squares (OLS) regression analyses examining associations between neighborhood factors and dichotomous physical activity (PA) outcomes.

			OLS regression results for continuous outcomes^a^		
PA outcome and neighborhood factor			Observations, n		Coefficient (95% CI)
**PA domain: transportation**					
	Walking environment	38		–31.45 (–217.21 to 154.30)	
	Aesthetic quality	39		–5.99 (–161.78 to 149.79)	
	Safety	38		47.58 (–91.97 to 187.13)	
	Social cohesion	35		–32.95 (–194.04 to 128.15)	
	Violence	33		20.30 (–156.13 to 196.73)	
	Activities with neighbors	38		102.70 (–97.72 to 303.12)	
**PA domain: leisure**					
	Walking environment	38		734.54 (–595.48 to 2064.57)	
	Aesthetic quality	39		558.27 (–538.28 to 1654.81)	
	Safety	38		724.93 (–266.29 to 1716.14)	
	Social cohesion	35		189.49 (–819.58 to 1198.57)	
	Violence	33		–1201.72 (–2537.39 to 133.95)	
	Activities with neighbors	38		–348.37 (–1778.59 to 1081.85)	
**PA intensity level: moderate to vigorous**					
	Walking environment	38		1934.56 (–418.61 to 4287.73)	
	Aesthetic quality	39		1662.83 (–273.18 to 3598.83)	
	Safety	38		1168.56 (–634.70 to 2971.83)	
	Social cohesion	35		593.57 (–1386.55 to 2573.70)	
	Violence	33		–1679.53 (–4229.44 to 870.37)	
	Activities with neighbors	38		509.87 (–2052.37 to 3072.10)	

^a^Controlled for age and level of education.

**Table 5:** Logistic regression analyses examining associations between neighborhood factors and dichotomous physical activity (PA) outcomes.^a,b^

PA outcome and neighborhood factor					Frequency, n (%)		OR (95% CI)
**PA domain: transportation**							
	Did not engage in transportation PA				16 (41)		1.00
	**Engaged in transportation PA**				23 (59)		
			Walking environment			1.60 (0.61-4.23)	
			Aesthetic quality			0.83 (0.38-1.78)	
			Safety			1.22 (0.60-2.47)	
			Social cohesion			1.45 (0.66-3.16)	
			Violence			1.17 (0.46-2.99)	
			Activities with neighbors			1.75 (0.59-5.16)	
**PA domain: leisure-time**							
		Did not engage in leisure-time PA			13 (33)		1.00
		**Engaged in leisure-time PA**			26 (67)		
			Walking environment			5.95* (1.49-23.74)	
			Aesthetic quality			2.45* (1.01-5.93)	
			Safety			3.30* (1.26-8.67)	
			Social cohesion			1.92 (0.81-4.55)	
			Violence			0.38 (0.13-1.10)	
			Activities with neighbors			0.67 (0.24-1.83)	
**Achieved national PA guidelines**							
		Did not meet PA guidelines			7 (18)		1.00
		**Met PA guidelines**			32 (82)		
			Walking environment			4.15* (1.13-15.18)	
			Aesthetic quality			6.43* (1.45-28.39)	
			Safety			2.53* (1.00-6.36)	
			Social cohesion			1.29 (0.53-3.15)	
			Violence			0.43 (0.16-1.20)	
			Activities with neighbors			0.99 (0.24-4.02)	

^a^Controlled for age.

^b^PA outcome variables for logistic regression analyses included engaging in transportation PA (ie, >0 metabolic equivalent [MET]-minutes/week of transportation PA) versus not engaging in transportation PA (0 MET-minutes/week of transportation PA), engaging in leisure-time PA (ie, >0 MET-minutes/week of leisure-time PA) versus not engaging in leisure-time PA (ie, 0 MET-minutes/week), and meeting national PA guidelines (ie, ≥500 MET-minutes/week of moderate-to-vigorous PA) versus not meeting national PA guidelines (ie, <500 MET-minutes/week of moderate-to-vigorous PA).

*Significant at a *P* value <.05.

## Discussion

### Principal Findings

This study explored PA patterns among first-generation Latina immigrants residing in the metropolitan area of Phoenix, Arizona, and the influence of perceived neighborhood environmental factors on these PA patterns. This work adds to the limited body of research on domain-specific PA patterns among first-generation Latina immigrants, with the majority of previous PA studies among Latinas having focused exclusively on leisure-time PA [34] and failing to differentiate by generational status. Given that first-generation immigrants likely have different lived experiences and lifestyle activities than Latinas who were born in the United States, work is needed in order to inform the development of PA interventions for this population.

Results showed that the majority of our sample (85%) reported PA levels that met or exceeded national PA guidelines. Domain-specific outcomes indicated that participants accrued the majority of their PA through domestic and household activities, with limited PA performed for leisure or transportation purposes. The perceived neighborhood environmental factors of walking environment, aesthetic quality, and safety were positively associated with engaging in leisure-time PA and meeting national PA guidelines. The neighborhood factors of violence, social cohesion, and activities with neighbors were not related to PA outcomes.

### Comparisons With Prior Work

Our finding that domestic and household activities accounted for the majority of PA performed reflects the outcomes of several previous studies examining PA patterns among Latinas [8,20] and supports the notion that Latinas, regardless of generational status, perform extensive caretaking and household activities as part of their daily routine. Likewise, low levels of leisure-time PA and a limited number of participants engaging in paid or unpaid work outside the home have also been previously reported among studies comprised predominantly of first-generation Latinas [19,20,33]. Leisure-time PA, in particular, has profound benefits for reducing cardiometabolic disease conditions [23,35,36]. Moreover, when compared with other PA domains (ie, work and transportation), leisure-time PA may be more amendable to change in the context of a PA intervention because it is within the volitional control of many Latinas. In future work, researchers should explore intervention strategies to increase leisure-time PA among first-generation Latinas. Such work may be key to reducing the disproportionate metabolic disease burden in this high-risk population.

Our examination of overall and intensity-specific PA patterns showed that our sample reported engaging in rather high levels of overall PA (ie, 4512 MET-minutes/week), with the majority of the PA being at a moderate intensity. We speculate that these high PA levels may be a result of overreporting, as this is a commonly reported occurrence with self-report PA measures [37]. Nicaise and colleagues [20] illustrated this issue in a previous study with low-income Latinas using the IPAQ and accelerometers. When assessing PA levels using the IPAQ, the authors found that 73% of Latinas met PA guidelines, with the bulk of the PA being accrued through domestic or household activities [20]—similar to the outcomes of our study. However, when examining PA levels with accelerometers, this percentage was reduced to 20%, suggesting that participants likely overestimated the intensity and the amount of PA accrued through domestic and household activities [20]. We speculate that a similar phenomenon may have impacted our study outcomes. Another key finding of our study was that our sample of Latinas reported limited vigorous-intensity PA, which also mirrors the outcome of several previous studies [18,20]. We hypothesize that this outcome was related to the limited amount of leisure-time PA reported by our sample, as vigorous PA is predominately achieved through purposeful exercise as opposed to daily activities [38].

When examining associations between neighborhood environmental factors and PA outcomes, linear regression models failed to show any significant associations. This might be due to the nonlinear patterns of the associations and a low statistical power due to the small sample size. However, logistic regression models revealed that the neighborhood factors of walking environment, aesthetic quality, and safety were associated with reported engagement in leisure-time PA and meeting national PA guidelines. These findings confirm previous studies showing that these factors are positively associated with PA engagement [19,39-42]. We also found it interesting that these 3 factors are ones that recent immigrants likely have limited control over (ie, these factors are primarily driven by public policies and allocation of local government funds). This outcome highlights the importance of considering all levels of the social ecological model, including public policy and urban design planning, when developing PA promotion interventions.

The lack of significant associations between the 6 neighborhood environmental factors and engagement in some versus no transportation PA in logistic models may be related to transportation PA being a necessity for daily activities rather an option (ie, participants may have had no other type of transportation). However, we did not include survey items asking participants about their primary mode of transportation, which is needed to draw a more precise conclusion on this outcome. Likewise, the lack of associations between social cohesion, violence, and activities with neighbors and PA was reported previously in a study among Latinx residing in Massachusetts [40], suggesting that these 3 factors may not be key determinants of PA engagement among Latinas. Future studies with larger sample sizes are needed before definitive conclusions can be drawn.

### Limitations of the Study

Limitations of our study include the use of a relatively small sample from a single metropolitan geographic region. The sample size was determined based on available funds, and the geographic region chosen was based on the population that the subsequent intervention would be developed to target. The small sample limited our ability to conduct additional subgroup analyses to further examine associations among duration of US residency (ie, ≤5 years versus >5 years), neighborhood environmental factors, and PA. Further examination of neighborhood characteristics and PA patterns based on duration of residency status in the United States would be interesting to explore in future work. Likewise, given that our sample comprised women residing in urban and suburban areas of metropolitan Phoenix, the study findings should not be generalized to residents residing in rural areas. Additional research with a larger, more geographically diverse sample is warranted to confirm and expand on our findings. Another limitation was that we relied exclusively on self-report measures to assess PA and neighborhood characteristics. Including an objective PA measure would have provided further context regarding the PA patterns of our sample. However, this was not possible because of the limited resources of the study. Likewise, preliminary data analyses resulted in 11 women being excluded from the study based on IPAQ scoring guidelines for either reporting nonplausible PA data (n=10) or providing incomplete data (n=1). Sensitivity analyses showed that these participants reported lower education levels than participants who provided valid data. This suggests that the IPAQ may not be appropriate for Latinas of lower education levels, further limiting generalization of the study findings. Additionally, perceived neighborhood characteristics may differ from objectively measured characteristics (eg, neighborhood characteristics measured using geographic information systems, local crime/violence data). Future research among first-generation Latinas should consider the use of both subjective and objective PA measures and neighborhood measures, as both types of measures provide important insights into the PA patterns and neighborhood characteristics of Latinas. A final limitation of this study is that it did not include items assessing BMI or health status. Such information would have provided additional insight on the generalizability of the study findings.

### Strengths of the Study

Despite its limitations, the current study has several strengths. First, this is one of few studies to examine domain-specific PA patterns among first-generation Latina immigrants, as most PA studies among Latinas have only included a measure of leisure-time PA [34] and failed to differentiate by generational status. Another strength of the study is that our sample was comprised of women from diverse countries of origin (ie, 69% from Mexico and the remainder from various Central and South American countries). The composition of our sample likely reflects the changing Latinx immigrant population in the United States (ie, immigration from Central and South American countries has increased in recent years, with immigration from Mexico slowly declining) [11].

### Conclusions

Findings suggest that many first-generation Latinas could benefit from a leisure-time PA intervention. Given that leisure-time PA has pronounced benefits for promoting positive health outcomes, intervention efforts targeting leisure-time PA may be an effective method for researchers and public health professionals to reduce obesity and diabetes health disparities among Latina immigrants. Such interventions should consider the neighborhood environments in which first-generation Latinas reside, as these factors will likely influence the types of PA promoted in an intervention. Results of this study will be used to inform development of a culturally tailored PA intervention for the reduction of metabolic disease risk among first-generation Latina immigrants.

## Appendix

Multimedia Appendix 1 Demographic questionnaire.

## Abbreviations

IPAQ: International Physical Activity Questionnaire
MET: metabolic equivalent
OLS: ordinal least squares
PA: physical activity
