# Supplementary material for: Physical Activity Patterns and Neighborhood Characteristics of First-Generation Latina Immigrants Living in Arizona: Cross-sectional Study
Source: JMIR Form Res. 2021 May 17;5(5):e25663. doi: 10.2196/25663 (PMC8167607; doi:10.2196/25663)
Supplement: Multimedia Appendix 1 [file formative_v5i5e25663_app1.docx]

**Demographic Questions**

**1. How old are you?**

________________________________________________________________

**2. Where were you born?**

- City ________________________________________________
- Country ________________________________________________
- Refuse to answer

**3. How long have you been living in the United States?**

- months ________________________________________________
- years ________________________________________________

**4. What language do you speak in your home most of the time?**

- English only
- English and Spanish
- Spanish only
- Other (please specify) ________________________________________________
- Refuse to answer

**5. Including money from all salaries/work, government assistance and (if applicable) unemployment, what is the total amount of money your household receives PER MONTH?**

- $0-1000
- $1001-2000
- $2001-3000
- $3001-4000
- >$4000
- Other amount (please specify) ________________________________________________
- Don't know
- Refuse to answer

**6. Last grade you completed in school**

- Less than 6th grade
- Completed elementary school (6th grade)
- Completed middle school (9th grade)
- Completed high school (12th grade)
- Some college
- College graduate or higher
- Other (please specify) ________________________________________________
- Don't know
- Refuse to answer

**7. What is your current marital status?**

- Single/No partner
- Married
- Living together (not married)
- Separated
- Divorced
- Widowed
- Other (please specify) ________________________________________________
- Refuse to answer

**8. Which of the following best describes your current employment status?**

- Unemployed or laid off and looking for work
- Unemployed and not looking for work
- Homemaker
- In school
- Retired
- Disabled, not able to work
- Other (please specify) ________________________________________________
